# Pathogenesis of Autoimmunity/Systemic Lupus Erythematosus (SLE)

**DOI:** 10.3390/cells14141080

**Published:** 2025-07-15

**Authors:** Shunichi Shiozawa

**Affiliations:** 1 Institute for Rheumatic Diseases, 60-14 Okuikeminamicho, Ashiya 659-0004, Japan; shiozawa@port.kobe-u.ac.jp; 2 Department of Medicine, Matsubara Mayflower Hospital, 944-25 Fujita, Katoshi 673-1462, Japan; 3 Department of Rheumatology, Graduate School of Medicine and Health Sciences, Kobe University, 7-10-2 Tomogaoka, Sumaku, Kobe 654-0142, Japan

**Keywords:** systemic lupus erythematosus (SLE), autoimmunity, pathogenesis, T follicular helper cells (Tfh cells)

## Abstract

SLE is characterized by the generation of a variety of autoantibodies including anti-dsDNA autoantibodies, causing damage in various organs. If autoimmunity is defined by the generation of a variety of autoantibodies against the self, SLE is the only disease to qualify. Identification of the SLE-causing factor must fulfill the following criteria: (i) the factor induces SLE, (ii) the factor is operating in active SLE and (iii) SLE heals after removal of the factor. All candidate factors are reviewed from this viewpoint in this review. As to the cause of SLE, high levels of interferon α can induce SLE; however, interferon α in most patients did not reach this high level. BAFF (B cell activating factor of the TNF family) is increased in SLE. BAFF itself induced some manifestation of SLE, whereas removal of interferon α or BAFF by an antibody (Ab) did not heal SLE. BXSB male mice with a duplicated *TLR7* gene develop SLE; however, the gene *Sle1* is also required for the development of SLE. In addition, *sanroque* mice develop a variety of autoantibodies and SLE; the *sanroque* mutation, which disrupts one of the repressors of ICOS, results in increased CCR7^lo^ CXCR5^+^Tfh cells, IL-21 and SLE. ICOS^+^T follicular helper (Tfh) cells increase in SLE and SLE-model (NZBxNZW)F1 mice, and the blockade of Tfh development ameliorated SLE, indicating the importance of Tfh cells in the pathogenesis of SLE. Self-organized criticality theory shows that SLE is caused by repeated infection, wherein SLE-inducing pathogens can vary individually depending on one’s HLA; however, the pathogen presented on HLA stimulates the T cell receptor (TCR) strongly beyond self-organized criticality. This stimulation generates TCR-revised, autoreactive DOCK8^+^Tfh cells, which induced a variety of autoantibodies and SLE. The SARS-CoV-2 virus is an example pathogen because SLE occurs after SARS-CoV-2 infection and vaccination. DOCK8^+^Tfh cells and SLE decreased after conventional or anti-DOCK Ab therapies. Thus, DOCK8^+^Tfh cells newly generated after repeated infection fulfill the criteria (i), (ii) and (iii) as the cause of SLE.

## 1. Introduction

**Figure 1:**
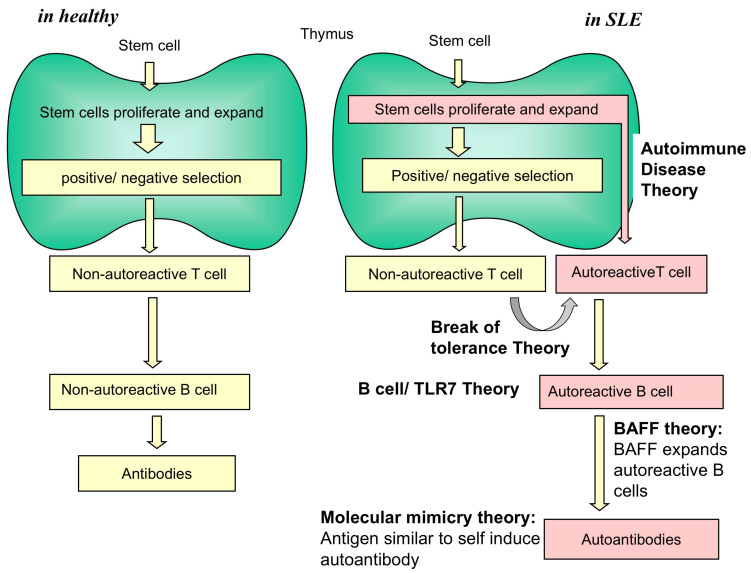
Normal immune response and diseased immune response passing through thymus. Left panel: Normal immune response. Right panel: Hypothetical response at the time of SLE. Autoimmune disease theory and other theories are shown.

Systemic lupus erythematosus (SLE) is a prototypical autoimmune disease, characterized by defects in the immune response and the generation of a variety of autoantibodies including anti-dsDNA autoantibodies, causing damage in various organs including kidney, skin and others [1,2]. Patients with SLE can experience complications from uncontrolled disease and from adverse effects and risks associated with glucocorticoids and immunosuppressive drugs that are used to treat lupus [3]. SLE is characterized by the generation of a variety of autoantibodies (in one case, over 100 varieties were found [2,4]) and in this respect, SLE is distinct from other ‘autoimmune diseases’ named by Mackay such as rheumatoid arthritis, dermatomyositis, systemic sclerosis, systemic vasculitis, Sjogren syndrome, etc. [5]. If ‘autoimmune disease’ or ‘autoimmunity’ were defined by the generation of a large variety of autoantibodies against self components, SLE would be the only disease to qualify. Indeed, previous investigators such as von Pirquet, Rossle or Ascoff recognized SLE to be distinct from other rheumatic diseases. However, Klemperer combined them altogether and named them Kollagen Krankheit, and then Mackey et al. again combined them altogether and named them autoimmune disease. The autoimmune disease theory of Mackay [5,6,7] states the following: (i) establishment of immune tolerance by deletion of self-reactive immunocytes during immunological maturation in early life; (ii) autoimmunity ensuing due to escape from such deletion of a clone of lymphocytes that could then proliferate in response to ‘self’ constituents as a pathogenic ‘forbidden’ clone; (iii) the origin of such ‘forbidden’ clones by somatic mutations among normal lymphocyte populations [5]; and (iv) undefined homeostatic processes exist for control of such forbidden clones (Figure 1).

Although this original thesis inspired a large number of studies, these have not actually identified nor recapitulated the autoimmunity caused by lymphocyte clones that have escaped deletion early in life and that proliferate as a pathogenic ‘forbidden’ clone in response to ‘self’ constituents. In other words, although many autoantibodies against self components have been found in SLE, the autoimmune disease SLE itself has never been experimentally induced by reaction against self constituents or forbidden lymphocyte clones reactive against self components.

In science and medicine, identification of a disease-causing agent requires three criteria: first, the causative agent should evoke the disease. Second, the agent should be found and operate in the disease. Third, the disease should subside after the removal of that agent.

To date, only the self-organized criticality theory [8,9] fulfills these three requirements, which proposes that SLE is caused by repeated infection with non-fatal, cytopathic pathogens, with or without the involvement of self peptides [8,9]. SLE-inducing pathogens can vary from person to person depending on one’s human leukocyte antigen (HLA), but the pathogenic antigen presented on HLA should be one that can maximally stimulate the T cell receptor (TCR) beyond self-organized criticality. What is critical is not the particular antigen, but rather the strength with which the antigen presented on HLA can stimulate the TCR. This stimulation will initially induce T cell anergy, but this anergy can then be broken by additional repeated stimulation of the TCR beyond its self-organized criticality. Repeated infection with a pathogen is usually less symptomatic because cytotoxic T cells are rendered into memory and thus can often escape medical notice. However, the SARS-CoV-2 virus infection and its vaccination are discernible and thus provide a notable example of this infectious model. SLE is significantly increased after repeated SARS-CoV-2 infection and its vaccination [10,11,12,13]; accordingly, the SARS-CoV-2 virus can be regarded as an SLE-inducing pathogen. SLE of model mice is also aggravated by repeated stimulation with SARS-CoV-2 spike protein [14].

There are many important studies on the pathogenesis of SLE, many of which have been overlooked in other review articles. Here, I review and attempt to critically assess such findings that may explain the pathogenesis of SLE.

## 2. Studies on B Cells, TLR7, TLR9, MyD88, BAFF, and IL-21

A number of studies have investigated how autoantibodies or autoreactive B cells can survive to exert their effects in vivo. These studies have demonstrated the importance of receptor editing to overcome allelic exclusion mechanisms, although they did not directly address how or why autoantibodies were generated [15,16]. In some of these experiments, tolerant B cells were found that could survive and generate autoantibodies despite allelic exclusion. Interestingly, these tolerant B cells were located in lymphoid areas outside of the germinal center and were activated with the help of Toll-like receptor 7 (TLR 7) signaling [16]. Tolerance has been shown to be a mechanism enabling autoantibody-producing B cells to survive. I believe these studies highlight the importance of inducing tolerance in antibody-synthesizing B cells and also relevant T cells that are responsible for the generation of autoantibodies.

The role of TLR7 was demonstrated in a mouse lupus model BXSB male mice. This mouse strain develops a severe form of murine lupus due to interactions between the y-linked autoimmune accelerator (*yaa*; Y chromosome-linked autoimmune acceleration) locus encoding *TLR7* on the Y chromosome and other autoimmune disease alleles in the BXSB genome [17]. The *Yaa* mutation was shown to be the consequence of a translocation from the telomeric end of the X chromosome onto the Y chromosome [17,18]. Induction of SLE required not only *yaa*-dysregulated B cells but also CD4 T cells, which did not necessarily carry the *yaa* mutation [19]. Introduction of the *yaa* mutation into nonautoimmune C57BL6J (B6) mice did not produce disease unless combined with other autoimmune-promoting genes [20,21,22]. For example, while B6.*yaa* mice themselves were not overtly autoimmune, the addition of *Sle1*, which contains the autoimmune-predisposing *Slam*/*Cd2* haplotype, results in the development of fatal SLE [22]. The B6.*sle1yaa* CD4 T cells were found to express the molecular signature of T follicular helper (Tfh) cells, notably expression of interleukin 21 (IL-21), inducible co-stimulator (ICOS) and programmed death-1 (PD-1), and these Tfh cells were associated with the development of severe pathology [22]. This finding of Tfh cells expressing IL-21, ICOS and PD-1 is compatible with the dedicator of cytokinesis 8 (DOCK8)-expressing Tfh (DOCK8^+^Tfh) cells of self-organized theory of autoimmunity [9].

Ablation of TLR 9 in the mouse MRL^lpr/lpr^ lupus model abrogated the generation of anti-DNA antibodies, but did not ameliorate clinical disease [23]. Ablation of TLR3, a receptor for dsRNA, did not abrogate the generation of autoantibodies to either RNA- or DNA-containing antigens [23]. Shlomchik’s group reported that TLR7 was required for anti-Sm autoantibody synthesis and TLR9 for anti-chromatin autoantibodies [24]. However, they showed that while TLR7 deficiency ameliorated disease, TLR9 deficiency actually exacerbated the disease in mice [24]. MRL^lpr/lpr^ lupus-prone mice that are genetically deficient in TLR7, TLR9, both TLR7 and TLR9, or myeloid differentiation primary response 88 (MyD88) showed that although TLR7 and TLR9 acted in parallel pathways on different subsets of autoantibodies, TLR9 suppressed the production of TLR7-dependent RNA-associated autoantibodies [24], and they mentioned that TLR- and MyD88-independent components are required for the induction of SLE in MRL^lpr^ mice [24]. Tilstra et al. used an inducible Cre-Lox recombinase system to selectively delete MyD88 in B cells after disease onset in MRL^lpr^ mice. This resulted in the amelioration of glomerulonephritis and renal interstitial inflammation and reduced autoantibody generation [25]. However, deleting MyD88 in dendritic cells (DCs) did not affect nephritis, despite the importance of DCs in renal inflammation, whereas MyD88 deletion in DCs did reduce dermatitis [26]. Fike et al. showed that IRF7^-/-^ in SLE-prone FcγR^-/-^ mice resulted in a significant decrease in renal disease, anti-dsDNA, anti-Sm and anti-nucleosome autoantibodies [27]. By making B cell-specific bone marrow chimeras where B cells were 100% deficient in IRF7, they also showed that anti-dsDNA and anti-Sm autoantibodies were significantly decreased [27]. Cosgrove et al. showed that TLR7 deficiency in CD19^+^ B cells of C57BL/6 mice yielded mild suppression of proteinuria, whereas B cell-intrinsic TLR7 expression enhanced renal disease and anti-RNA, but not anti-Sm Ab or anti-nucleosome autoantibodies in TLR-9-deficient MRL/lpr mice [28]. A slight decrease in proteinuria does not indicate amelioration of disease because renal pathology does not correlate with proteinuria. B cell-specific IRF7 deficiency also did not or mildly reduced Tfh cells that are a characteristic feature in SLE [27,28]. Thus, IRF7 did not induce SLE. Few SLE patients with heightened IRF7, except for a similar patientwith mutation, were reported [29] and thus, TLR7, TLR9 or MyD88 does not fulfill criteria as a disease-causing agent.

Transgenic mice overexpressing BAFF (B cell activating factor of the TNF family) develop autoimmune-like manifestations such as high levels of rheumatoid factors, circulating immune complexes, and immunoglobulin deposition in the kidneys [30,31,32]. Gross et al. showed that BAFF and anti-dsDNA antibody levels were increased in BAFF-transgenic mice and also in mouse (NZBxNZW)F1 and MRL^lpr^ models of SLE [31]. However, as the authors themselves pointed out [30], few cases of SLE organ disease were observed in BAFF transgenic mice [30,31] or BAFF transgenic mice without T cells [32] other than some cases of disease in the kidney and salivary glands. Anti-DNA autoantibodies were increased in only a minor population of the mice [30], while the same authors showed that anti-dsDNA autoantibodies were raised in the mice. However, other varieties of autoantibodies typically found in SLE patients were not observed [30,31,32]. It has been proposed that BAFF promotes autoantibody production by rescuing low-affinity, autoreactive transitional B cells from deletion via signals dependent on TACI (transmembrane activator and CAML interactor) [33]. BAFF expression levels are increased in patients with SLE and serum levels of BAFF correlate with anti-DNA Ab levels [34,35,36]. However, conflicting results have also been reported with regard to the relationship between serum BAFF levels and SLE disease activity [34]. Clinical anti-BAFF Ab treatment added to conventional therapy showed no effect [37] or only a modest effect in decreasing lupus flare rates over long periods of time [38,39,40,41]. It was clear that anti-BAFF Ab treatment by itself did not entirely ameliorate SLE and thus, BAFF is not the cause of SLE, although it aggravates SLE disease activity.

The cytokine IL-21 is a major product of chemokine receptor 5 (CXCR5)^+^ICOS^+^CD4^+^ Tfh cells as well as of CD4^+^CXCR5^lo^ICOS^+^ extra-follicular Tfh cells, which are involved in the extrafollicular differentiation of B cells [42]. IL-21 signaling to B cells is essential for inducing SLE in mouse lupus models [43,44]. In one autoimmune mouse model, IL-21-producing Tfh-like cells were found in lymphoid extra-follicular sites [42], a finding consistent with our self-organized criticality theory that SLE-inducing and IL-21-producing DOCK8^+^ Tfh cells exist in the splenic red pulp of SLE patients [9]. Wang et al. showed that IL-21 and IL-21^+^ Tfh cell numbers correlate with SLE disease activity [45]. Ozaki et al. showed that IL-21 signaling to B cells is essential for the development of all classical disease manifestations seen in BXSB.*Yaa* SLE mouse models [46].

## 3. Autoantibody Studies

Sequence analysis of anti-DNA antibodies has shown that IgG anti-DNA autoantibodies in various autoimmune lupus mouse models and in human SLE are oligoclonal and somatically mutated, which may reflect antigen selective pressure operating during their development, suggesting that both antigen and antigen-specific helper T cells drive the autoimmune response [47,48]. Pathogenic anti-dsDNA autoantibodies that contribute to lupus nephritis in humans as well as in murine models of the disease had undergone both somatic mutation and heavy chain class switching, suggestive of antibodies found in an antigen-activated response [49,50]. A foreign antigen was proposed to be a driving force in generating these autoantibodies, since housing lupus-prone (NZBxNZW) F1 mice in a germ-free facility greatly delayed the onset and reduced the severity of autoimmune disease [49,50,51]. The TLR7 studies discussed above also demonstrated a requirement for CD4 T cells, likely Tfh cells.

These studies thus show that stimulation with an antigen is required for generating autoimmunity and also that the autoreactivity in helper T cells is responsible for autoantibody synthesis and the pathogenesis of SLE, a mechanism that is compatible with the self-organized criticality theory of autoimmunity [8,9].

## 4. T Cell Studies

Studies on T cell tolerance have shown that helper T cell tolerance is much more profound and sustained than B cell tolerance [52]. Weigle showed that tolerance to foreign serum albumins could be broken by immunization with a related serum albumin, resulting in the production of an antibody reacting against both the immunogen and the tolerogen [53]. Lin et al. showed that immunization of mice with a non-self protein (human cytochrome c) could induce B cells to present self antigens (mouse cytochrome c) to autoreactive T cells in an immunogenic form, resulting in the generation of antibodies against epitopes shared by both the immunizing (human cytochrome c) and the tolerated protein (mouse cytochrome c) [54]. The authors suggested that this mechanism of breaking T cell self tolerance could account for the role of foreign antigens in breaking not only B cell but also T cell self tolerance, which would lead to sustained autoantibody production in the absence of the foreign antigen. This type of immunization, termed “molecular mimicry”, was achieved by antigen stimulation in the presence of a complete Freund adjuvant. The use of an adjuvant is artificial, but the use of an adjuvant probably played a similar role as the repeated stimulation with an antigen that we showed [8,9].

T follicular helper cells (Tfh) cells, specialized for providing help to B cells, could play a major role in GC reaction and selection of high-affinity B cells. Tfh cells and their precursors secrete large amounts of IL-21, a γc-family cytokine that potently promotes the growth, differentiation and class-switching of B cells [55]. In GCs, IL-21 produced by Tfh cells plays a critical role for the generation and survival of GC B cells [56]. Excessive Tfh cell generation together with excessive GC formation has been shown to lead to the expansion of autoreactive B-cell clones, which correlate with lupus disease activity and/or serum autoantibody titers [57]. These circulating CCR7^lo^PD-1^hi^ Tfh cells should include the DOCK8^+^Tfh population that we have proposed to be the causative agent of SLE [9] and their numbers are increased in correlation with SLE disease activity [58]. These CCR7^lo^PD-1^hi^ Tfh cells were further shown to be responsible for protective antibody responses during infection, vaccination and autoantibody synthesis [58]. Importantly, these Tfh cells did not express CCR7, and therefore should not be able to migrate into GC, and thus remain in the extra-follicular space. Chauveau et al. showed that CCR7 was required for T cell entry from the vasculature into T cell zones [59]. We showed that the disease-causing DOCK8^+^Tfh cells were located in the red pulp of the spleen of SLE patients [9]. The extra-follicular space is a suitable location for encountering circulating antigens and nursing autoreactive B cells [60]. Simpson et al. showed that circulating CXCR5^+^PD-1^hi^ Tfh cells, which should also include DOCK8^+^Tfh cells, were increased in SLE in correlation with the diversity and titers of autoantibodies and the severity of end-organ involvement [61].

Akama-Garren et al. clearly showed that autoreactive follicular T cells are necessary and sufficient to break B cell tolerance and initiate autoantibody formation [62]. They showed that one Tfh cell having an autoreactive TCR could stimulate B cells to produce several different autoantibodies. It is not that a single autoreactive Tfh TCR is sufficient to drive polyclonal humoral autoimmunity, as a review by Wellford and Schwartzberg wrote [63]. I interpret their findings to mean that several different autoantibodies can be generated due to partial structural identity or similarity to the autoantigen, and it is not epitope spreading.

CCR7^lo^ CXCR5^+^ Tfh cells, which should include DOCK8^+^Tfh cells [9], are increased both in the *sanroque* mouse model of SLE and human SLE and they serve to facilitate autoantibody formation [58]. Vinuesa et al. discovered a new ubiquitin ligase family member, Roquin, that is an essential negative regulator of Tfh cells [64]. They showed that the *sanroque* mutation disrupts a repressor of ICOS, resulting in excessive Tfh cell and IL-21 production. These *sanroque* mice develop high titers of autoantibodies and exhibit a pattern of pathology consistent with lupus [64]. This CCR7^lo^CXCR5^+^Tfh population should include the DOCK8^+^ Tfh cells that we found to be able to cause SLE [9]. Other studies have also shown that ICOS^+^ T cells, which should also include DOCK8^+^ Tfh cells, are expanded in both the (NZBxNZW)F1 mouse model of SLE and human SLE patients, while blockade of this pathway ameliorated disease severity in a murine model of SLE [65,66].

The involvement of Tfh cells in the pathogenesis of SLE is supported by genetic susceptibility studies, including genome-wide association studies. In addition to genes expressed by Tfh cells such as *IL21* or *IL21R*, these studies identified numerous susceptibility loci related to the differentiation of human Tfh cells, such as *IL12A* (a subunit of IL-12), *STAT4* (a transcription factor involved in IL-12 signaling), *TYK2* (an IL-12 signaling molecule), *TNFSF4* (encoding OX40L), and the transcription factors *IRF5* and *IRF8* [56].

## 5. Studies on Interferon α

In a critical review, Crow et al. [67] referenced an older study showing that exogenous interferon α could induce glomerulonephritis when injected into newborn mice [68]. Further, Adam et al. showed that mice injected with interferon α (IFNα) showed delayed growth, decreased survival and enhanced severity of glomerulonephritis [69]. IFNα also increased the levels of serum anti-ssDNA and anti-soluble nucleoprotein antibodies in these mice. Using an inducible IFNα transgenic mouse model, Akiyama et al. showed that conditional upregulation of IFNα alone can induce SLE symptoms in mice otherwise not prone to autoimmunity. The symptoms included the generation of serum immune complexes, autoantibodies including anti-dsDNA antibody, and classical organ disease manifestations typical of SLE, such as immune complex-deposited glomerulonephritis, classical splenic onion-skin lesion, alopecia, epidermal liquefaction, positive lupus band test of the skin, and alopecia [70]. It was shown that higher IFNα levels were responsible for the induction of SLE and, in particular, for the organ manifestations such as lupus skin disease and nephritis. We showed in SLE patients that much more high levels of IFNα were responsible for the induction of lupus psychosis [71].

It should be noted here that SLE symptoms required higher levels of biologically active IFNα [70,71]. IFNα is easily fragmented in sera or in body fluids and loses its biological activity [72], whereas our radioimmunoassay could measure biologically active IFNα [71,72]. However, previous investigators claiming that IFNα induces SLE did not take into account of the levels or bioactivities of IFNα, which may have led to the false conclusion that IFNα causes SLE. In some cases, IFNα ameliorates autoimmunity [73,74]. Hron and Peng showed that deficiency of the IFN receptor type I in MRL/lpr mice worsened lymphoproliferation, autoantibody production, and end organ disease [74].

Baccala et al. indicated that the pathogenic process in SLE must require high IFNAR availability and signaling [75]. This would explain the reason why only a minor fraction of IFNα-treated patients succumb from SLE [76,77,78,79,80], why only lupus-prone mice, but not mice without genetic predisposition to autoimmunity, develop SLE upon continuous exposure to high amounts of IFNα [81,82,83], and why type I IFNα sometimes showed protective effects on SLE [73,74]. Ronnblom et al. showed that SLE was developed in 1 out of 135 patients with malignant carcinoid tumors treated with IFNα of an extremely high dose daily of 3~6 × 10^6^ U, 3 times a week [78], which means that substantially higher IFNα was required for SLE to develop, and that even under a higher dose of IFNα, only a few individuals succumb from SLE.

An earlier study measuring serum IFNα by bioassay showed that IFNα was detectable in the sera of 76.6% (31/41) of active SLE patients, but IFNα titers in all except one of the patients were less than 128 IU/mL [84]. The one exception was a 33 y.o. female SLE patient who had high biological IFNα levels at 512 IU/mL and who had joint involvement, rash, pancytopenia, oral ulcers, weakness, fatigue, and anorexia [84]. Proteinuria and hematuria were also present, and focal membranous and proliferative glomerulonephritis was found by a renal biopsy. Serologic studies in this patient showed decreased C3 and increased anti-DNA antibody [84]. During the ensuing year, this patient was treated with prednisone and hydroxychloroquine, which led to a gradual improvement in symptoms and serologic markers. During this period, the patient’s IFNα titers dropped from 512 U/mL to less than 16 U/mL. The SLE of this particular patient could be caused by upregulated IFNα. Preble et al. showed that IFNα was detectable in the sera of 47.1% (65/138) of SLE patients and their titers were all below 128 IU/mL, with 60% of them below 64 IU/mL [85]. Von Wussow et al. showed that IFNα was detectable in the sera of 15% (9/61) of SLE patients by bioassay with titers between 6 and 40 IU/mL, whereas IFNα was detectable in 28% (17/61) of patients by radioimmunoassay [86]. In fact, in most SLE patients, the IFNα levels are not high enough to induce SLE [9,70,71].

Efficacy of anti-IFNα receptor therapy was minor or moderate, reducing SLE disease activity without offering a cure [3,87,88,89,90,91]. Several reports have shown that excessive type I interferon levels may cause Aicardi–Goutières syndrome (AGS), a disease distinct from SLE [92,93].

As mentioned earlier, three criteria to identify a disease-causing agent are as follows: (i) the agent should evoke the disease, (ii) the agent should be found and operate in the disease, and (iii) the disease should subside after the removal of that agent. For IFNα, while criteria (i) is fulfilled as our study showed that transgenic higher levels of IFNα induce SLE in mice [70], criteria (ii) is not fulfilled as sufficiently high levels of IFNα are generally not attainable. Criteria (iii) is also not fulfilled because SLE is not cured after the removal of IFNα.

## 6. Self-Organized Criticality Theory of Autoimmunity; Infection Causes SLE

SLE is caused by both genetic predisposition and environmental stimulation [94,95].

**Figure 2:**
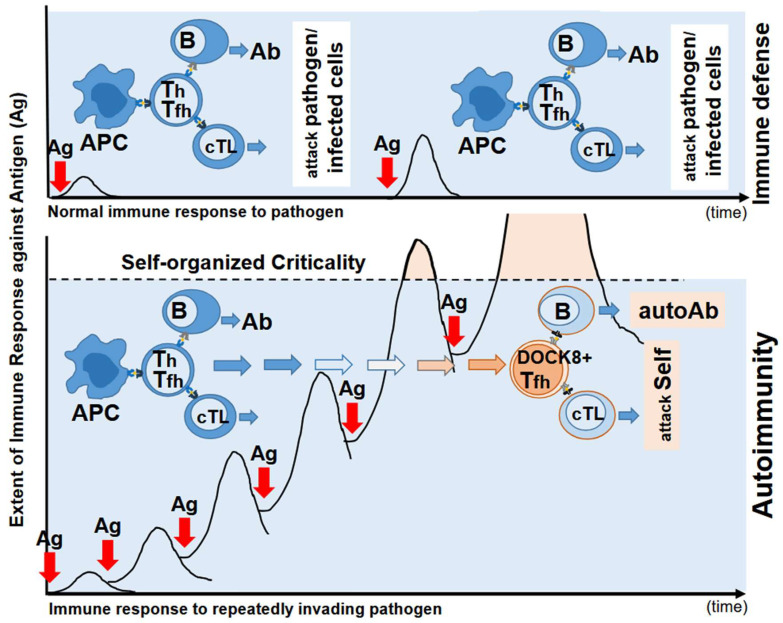
Self-organized criticality theory of autoimmunity [8,9]. Upper panel: Normally, when antigen (Ag) invades, antigen-presenting cells such as dendritic cells (DCs) present Ag to T cells. T helper cells (Th) or T follicular helper cells (Tfh) then stimulate B cells to produce antibodies (Abs) against invading Ag and stimulate to become cytotoxic T cells (cTL), which destroy Ag-infected cells. When Ag invades again, the same but exaggerated response will take place. Lower panel: When Ag invades repeatedly in a frequent fashion, immune response will be exaggerated. However, when stimulated further, cellular responses cease and T cells are tolerized. When Ag continued to invade further beyond the self-organized criticality of the immune system response, tolerized T cells are re-activated and become T follicular cells (Tfh cells) and their TCR will be revised. This TCR revision is random, and thus autoreactive T cells are included in this. This TCR revision may or may not be random because previously unread early TCR genes are read at this TCR revision [9]. The T cells that passed through TCR revision then educate B cells to produce autoantibodies.

Repeated stimulation of TCR with an antigen to levels that surpass the host’s steady-state response, i.e., self-organized criticality, induced SLE in mice normally not prone to autoimmunity [8]. In these experiments, repeated stimulation generated Tfh cells expressing the guanine nucleotide exchange factor DOCK8 on the cell surface [9]. These DOCK8^+^Tfh cells underwent TCR re-revision in the periphery, induced a variety of autoantibodies, and subsequently overstimulated CD8 T cells, driving them to become antigen-specific cytotoxic T lymphocytes (CTL). These CTLs could be further matured by antigen cross-presentation, after which they caused autoimmune tissue injury characteristic of SLE [8,9,96] (Figure 2).

The immune response is a system response. The system components are separable from biochemical characteristics, as different chemistries can produce the same system properties. The steady-state immune response is maintained by complex mutual interactions among multiple components such as immune cells and cytokines. In the immune system, the input signal, i.e., antigen stimulation, should be characterized by its strength, timing, duration, and frequency. While the system normally maintains a steady state in response, there is a stability limit in systems response, a self-organized criticality. In immunology, the self-organized criticality corresponds to tolerance or anergy. The response to self-organized criticality in the immune response has not been widely appreciated in immunology and thus has been studied by only a few groups. Such studies have shown that upon repeated encounters with peripherally expressed antigens, such as mouse mammary tumor virus (Mtv-8) or influenza hemagglutinin (HA), the CD4 T cells reactive against these antigens are deleted or anergized [97,98]. Anergized CD4 T cells acquire a T follicular helper-like phenotype, and their TCR is revised [97,98,99,100]. Because these cells no longer react against the same antigen and reside in a restricted space, i.e., the germinal center (GC), further immune responses cease [98]. However, Adler’s group showed that such cells can proliferate again when stimulated further [97]. However, what happens subsequently was not studied by any investigators.

The system response is determined by the strength, timing, duration, and frequency of stimulus, but not the chemical characteristics of the antigen, and thus, the systems response is driven by an antigen plus HLA (antigen presented on HLA). For this reason, SLE-inducing antigen differs from person to person depending on one’s HLA. Thus, HLA is a major genetic predisposition factor. If the immune system is further stimulated beyond self-organized criticality by an ‘immunogenic form’ of antigen such as a virus, anergized T cells begin to proliferate and result in the generation of autoreactive DOCK8^+^Tfh cells that have undergone TCR revision [8,9].

In view of the immune response against pathogens, the immune system protects the host from infections primarily by eliminating invading pathogens, but overly exuberant immune responses can also cause collateral damage to the host through immune effectors, and thus some non-lethally harmful microbes are afforded to repeatedly invade [101,102]. Such pathogens or the endogenous antigen whose sequence might be integrated into viruses as an immunogenic form can repeatedly stimulate the TCR. Recent COVID-19 infection studies have shown that repeated SARS-CoV-2 virus infection or its vaccination can induce SLE [10,11,12,13]. Thus, SARS-CoV-2 virus is an example of an SLE-inducing pathogen in our self-organized criticality theory of autoimmunity as this virus can induce SLE [10,11,12,13] if presented on a susceptible HLA over time [8,9]. HLA is therefore a major genetic predisposition factor [103]. SLE of model mice is also aggravated by repeated stimulation with SARS-CoV-2 spike protein [14]. Another example pathogen is human papillma virus 16 and its causal relationship with SLE has been established by using the two-sample bidirectional Mendelian randomization method [104,105]. The HPV L1 protein shows a significant overlap in peptide sequences with various human proteins, including those recognized as autoantigens in individuals with SLE such as lupus Ku autoantigen proteins p86 and p70 or natural killer cell IgG- like receptors [104].

Importantly, DOCK8^+^Tfh cells possess classical Tfh characteristics but are excluded from the GC because they are CCR7^lo^ and therefore are present in increased numbers in the splenic red pulp and peripheral blood of active lupus patients [9]. Splenic sinus, red pulp, is a suitable location to encounter circulating antigens and for nursing autoreactive B cells derived from bone marrow. Some of these shuttle between the marginal and follicular zones via expression of CD138, and undergo class-switch recombination to differentiate into plasma cells [42,60,106,107]. In our model [9], germinal center B cells and matured B cells were indeed increased, which are characteristic of autoantibody-producing B cells found in SLE [108,109]. In the red pulp, DOCK8^+^CD4 T cells could also meet the red pulp macrophages that are highly efficient at cross-presenting antigens to T cells and maturing CTLs [110], which are essentially important for the generation of lupus tissue injuries, as shown previously [8,9,96].

Therefore, repeated stimulation with an ‘immunogenic’ antigen, either exogenous or endogenous, generates DOCK8-expressing Tfh cells, which then induce a variety of autoantibodies and SLE. Self-organized criticality theory in which induction of SLE by repeated stimulation with viral ‘‘immunogenic forms’’ of an autoantigen is compatible with the molecular mimicry theory of autoimmunity [111,112]. However, for this mechanism to be valid, the host’s steady-state immune response must be compromised beforehand, as we showed [8,9]. SLE is unique in that a large variety of autoantibodies, sometimes exceeding 100 types, are generated in one individual [4,113], and thus generation of autoantibodies and SLE as the result of TCR re-revision at the periphery seems more straightforward and logical than a mechanism involving the reactivation of forbidden clones.

Self-organized criticality theory also showed that DOCK8^+^Tfh cells in peripheral blood and in the splenic sinus both decline after conventional therapy in patients with SLE [9]. Autoantibodies and lupus organ lesions were significantly decreased by anti-DOCK8 antibody in mice, including SLE-model (NZBxNZW) F1 mice [9]. The transfer of DOCK8^+^Tfh cells to healthy recipient mice resulted in the generation of a variety of autoantibodies and organ diseases in the kidney, skin, lung, and spleen, indicating that DOCK8^+^Tfh cells can induce SLE [8,9]. The DOCK8^+^Tfh cells newly generated after repeated infection with pathogens fulfill the criteria of a disease-causing agent: (i) DOCK8^+^Tfh cells can induce SLE, (ii) DOCK8^+^Tfh cells are increased in active SLE, and (iii) SLE subsides after antibody-mediated removal of DOCK8^+^Tfh cells in mice or after decreasing DOCK8^+^Tfh cells by conventional therapy in the patients.

In summary, SLE is caused by DOCK8^+^Tfh cells generated after repeated TCR stimulation by immunogenic forms of pathogens, i.e., infection, to levels that surpass the system’s self-organized criticality. The pathogen can be either exogenous or endogenous and is presented with HLA. It is the strength of the TCR stimuli (antigen plus HLA), and not the particular pathogen, that is critical for inducing SLE, and thus the pathogen may differ among individuals depending on one’s HLA. The SARS-CoV-2 virus is an example of this pathogen.

## 7. Concluding Remarks

Autoimmunity, as termed by Mackay [5], refers to an immune attack against the self and diseases caused by the immune attack against the self. Extensive studies have been carried out to clarify how an immune attack against the self can cause autoimmunity. These studies have been largely unsuccessful, and instead supported the idea that the immune system basically does not attack the ‘self’.

From an evolutionary viewpoint, reproduction is the key for evolutionary success, and thus people, especially women, need to survive until at least the age of reproduction. Possibly for this reason, the immune response against pathogens is stronger in women [114,115]. An immune attack against the self is certainly disadvantageous for evolution and thus should be genetically selected against during evolution. A genetically non-coded mechanism, including autoimmune disease theory, should not operate even in disease, and thus the autoimmune disease theory is invalid. Autoimmunity is induced by repeated infection with non-fatal, cytopathic pathogens, with or without self-peptide sequences [8,9]. Whether or not repeated stimulation by pathogen surpasses the self-organized criticality of the immune response is of fundamental importance. Self-organized criticality theory is exemplified by SARS-CoV-2 virus infection and its vaccination [10,11,12,13,14].

## 8. Patents

Patent#7037198 (patented 8 March 2022, Japan); patent on application #PCT/JP2018/007012; patent on application #2023-129-483 (patented 7 January 2025, Japan); patent on application #PCT/JP2024/027490.

## Abbreviations

AGS	Aicardi-Goutières syndrome
Ab	Antibody
BAFF	B cell activating factor of the TNF family
BXSB/MpJ mice	A recombinant inbred mouse line produced from an intercross between C57BL/6J (B6) and SB/Le mice
CCR7	Chemokine receptor 7
CTL	Cytotoxic T lymphocytes
CXCR5	Chemokine receptor 5
DC	Dendritic cell
DOCK8	Dedicator of cytokinesis 8
GC	Germinal center
*gld*	Generalized lymphoproliferative disease gene
HA	Hemagglutinin
HLA	Human leukocyte antigen
ICOS	Inducible co-stimulator
IFN	Interferon
IRF	IFN regulatory factor
IL	Interleukin
*lpr*	Lymphoproliferation gene
MtV-8	Mouse mammary tumor virus-8
MyD88	Myeloid differentiation primary response 88
NZW mice	New Zealand White mice
NZB mice	New Zealand Black mice
OX	Orexin receptor
PD-1	Programmed death-1
SARS-CoV-2	Severe acute respiratory syndrome Corona virus 2
strain SB/Le	Strain of mice homozygous for the mutant genes beige (bg), satin (sa), and white-bellied agouti (A^w^)
*Slam*	Signaling lymphocytic activation molecule gene
STAT4	Signal transducer and activator of transcription 4
TACI	Transmembrane activator and CAML (calcium-modulating cyclophilin ligand) interactor
TCR	T cell receptor
Tfh cell	T follicular helper cell
TLR	Toll-like receptor
*TNFSF4*	Tumor necrosis factor superfamily, member 4
Tyk	Tyrosine kinase
*yaa*	Y chromosome-linked autoimmune acceleration gene
